# Trajectory Generation for Flexible-Joint Space Manipulators

**DOI:** 10.3389/frobt.2022.687595

**Published:** 2022-03-31

**Authors:** David S. Carabis, John T. Wen

**Affiliations:** ^1^ Department of Mechanical, Aerospace, and Nuclear Engineering, Rensselaer Polytechnic Institute, Troy, NY, United States; ^2^ Department of Electrical, Computer, and Systems Engineering, Rensselaer Polytechnic Institute, Troy, NY, United States

**Keywords:** space robot, flexible joint arm, trajectory generation, manipulation, free-flying base

## Abstract

Space manipulator arms often exhibit significant joint flexibility and limited motor torque. Future space missions, including satellite servicing and large structure assembly, may involve the manipulation of massive objects, which will accentuate these limitations. Currently, astronauts use visual feedback on-orbit to mitigate oscillations and trajectory following issues. Large time delays between orbit and Earth make ground teleoperation difficult in these conditions, so more autonomous operations must be considered to remove the astronaut resource requirement and expand robotic capabilities in space. Trajectory planning for autonomous systems must therefore be considered to prevent poor trajectory tracking performance. We provide a model-based trajectory generation methodology that incorporates constraints on joint speed, motor torque, and base actuation for flexible-joint space manipulators while minimizing total trajectory time. Full spatial computer simulation results, as well as physical experiment results with a single-joint robot on an air bearing table, show the efficacy of our methodology.

## 1 Introduction

Robotic manipulation is an attractive technology for many on-orbit applications. These applications include satellite servicing, space structure construction, and debris management (Yoshida, 2003; Moosavian and Papadopoulos, 2007; Ogilvie et al., 2008; Tasker and Henshaw, 2008; Flores-Abad et al., 2014). The use of autonomous robots for these tasks (with on-ground instruction) can help to reduce the need for astronaut resources, as well as pave the way for widespread adoption by industry and governmental space agencies alike.

Space manipulators are often lightweight, meaning they have limited motor torques and are flexible. This type of design is often necessary to meet payload requirements (Yamano et al., 2000). Link flexibility may arise from long lightweight links, while joint flexibility may arise from large gear ratios and harmonic drives (Flores-Abad et al., 2014). However, joint flexibility due to harmonic drives often has lower resonant modes than link flexibility, and is generally the dominant source of flexibility when considering captured object transport (Alberts et al., 1992; Flores-Abad et al., 2014). One example is the shuttle Remote Manipulator System (Canadarm), where joint flexibility generates the first brakes-on natural frequency at 0.03 Hz, while the lowest resonate mode due to link flexibility is at about 23 Hz (note that this data is for the typical payload grasping configuration, with grasped payload) (Alberts et al., 1992).

**FIGURE 1:**
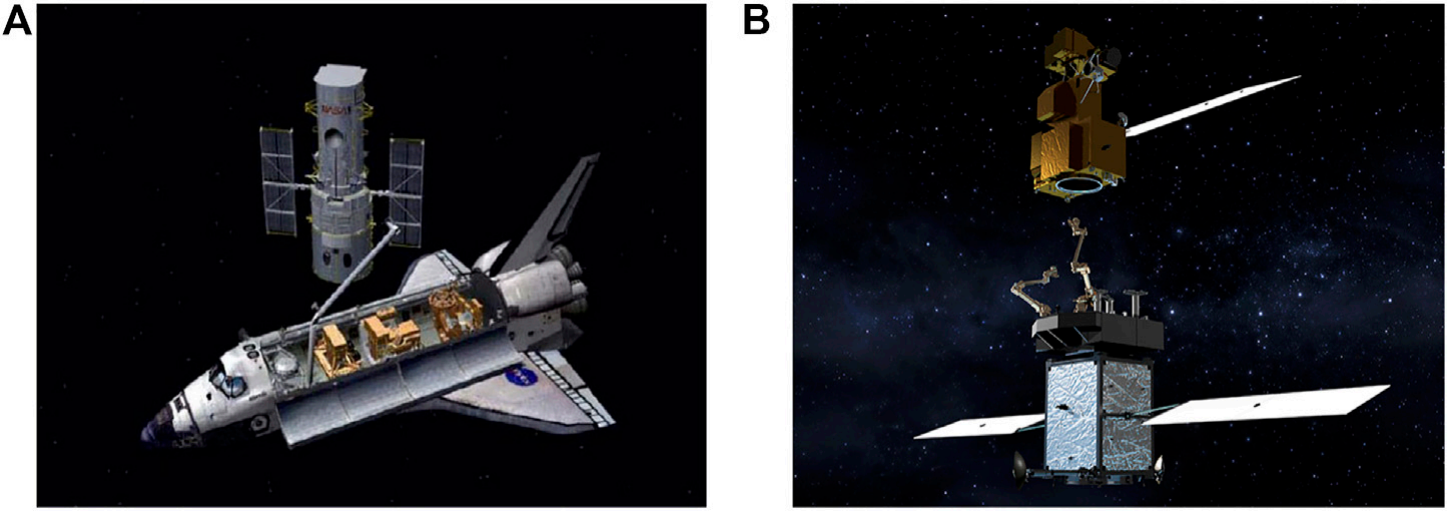
Examples of lightweight articulated manipulators for massive object capture and transport: **(A)** the Hubble telescope during a repair mission, **(B)** satellite servicer capturing a satellite. Photo credit: NASA.

Many objects that would be manipulated on-orbit are also massive, such as a satellite in a servicing application. One example is the Hubble Space Telescope, which has a mass of 12,000 (Garner, 2018). Servicing applications are illustrated in Figure 1. The combination of limited motor torque, joint flexibility, and massive payload can lead to accuracy reductions, excessive vibrations, and trajectory following difficulties (Flores-Abad et al., 2014). Dangerous scenarios, such as object collision with the manipulator base or self-collision, may arise.

Regardless of these limitations, negligible gravitational effects allow such arms to transport massive objects. Two flight examples of lightweight arms are Canadarm and Canadarm 2 (also known as the shuttle and International Space Station (ISS) arms) (Scott et al., 1993; Ma et al., 1997). However, these arms are often teleoperated by astronauts during large object transport, such as when the SpaceX Dragon was docked at the ISS (Kauderer, 2012). Visual feedback can then be used by astronauts to avoid oscillatory behavior and significant trajectory following error. This capability is not available for ground teleoperation scenarios, where significant visual time delay is present (Flores-Abad et al., 2014).

In spite of these challenges, successful on-orbit capture and transportation tasks have taken place using limited ground control. Capture and docking of a cooperative satellite has been demonstrated by NASDA’s ETS-VII satellite (Yoshida, 2003). Autonomous capture has been demonstrated by the DARPA Orbital Express Demonstration System (Flores-Abad et al., 2014). However, a key aspect of these two operations is a relatively small target object mass compared to the robot base [400 kg–2,500 kg for ETS-VII (Kasai et al., 1999); 226 kg–952 kg for the Orbital Express Demonstration System (Bell, 2020)]. Both operations also involved cooperative target satellites (i.e., satellites designed for manipulation).

Most recently, Northrop Grumman’s MEV-1 satellite successfully docked with a non-cooperative satellite to extend lifetime after the target satellite’s fuel was depleted. MEV-1 rigidly docked with the target satellite and now acts as a propulsion stage for both satellites, rather than refueling and restoring propulsion (Chang, 2020; Northrop Grumman Space Logistics, 2020). This differs from robotic docking and servicing in several key ways.

First, MEV-1 utilizes an apogee kick thruster cone for docking (Northrop Grumman Space Logistics, 2020). This feature is present on approximately 80% of satellites in geosynchronous orbit (Hall, 2019), meaning that there are satellites that cannot be serviced via this method. Furthermore, this feature may be less prevalent in satellites in low Earth orbit. The fact that MEV-1 acts as a propulsion stage also means that only one target satellite can be helped at a time, as opposed to a refueling operation where both satellites must only remain docked for the duration of the refueling procedure. The presence of a robotic manipulator also opens the door to other servicing and repair procedures, such as replacing satellite components (Flores-Abad et al., 2014).

This does of course beg the question of why cone docking and a robotic arm are not used in tandem. While this is a potential solution for some scenarios, there are certain satellites that would not be able to be serviced, as stated above. This setup would also require additional launch mass for a servicing satellite. Cone docking also does not address the manipulation of other massive objects in space, such as components of space structures and scientific samples from asteroids.

Even with these successful on-orbit operations, robotic manipulation in space remains an open research area due to challenges with the manipulation of massive non-cooperative objects (here defined as objects not designed for grasping and manipulation) (Flores-Abad et al., 2014). Modeling of flexible joint space manipulators has been addressed. Some existing models have simplified the system by assuming the motor kinetic energy is only due to relative motion about the motor axis (Spong, 1987; Yu and Chen, 2014), while other models have incorporated the intertial coupling of the motors and arm links (Ulrich et al., 2012; Nanos and Papadopoulos, 2015). Furthermore (Ulrich et al., 2012), has incorporated nonlinear spring and friction models in the flexible joints. Models with both flexible joints and flexible links have also been investigated (Yu and Chen, 2014).

Flexible joint control has also been addressed (Spong, 1987; De Luca et al., 1989; Spong, 1989; Sun et al., 2018; Yin et al., 2018), including for space manipulation with a floating base. Control utilizing an additional link-side torque sensor and an inner torque feedback loop has been applied to a single flexible joint in simulation (Ferretti et al., 2005). The limitation of needing an additional link-side torque sensor was later addressed by (Ulrich and Sasiadek, 2012a), where an extended Kalman filter was used to estimate the link-side state for a 2 degree-of-freedom (DOF) robot in simulation. Adaptive control has been utilized to reduce trajectory tracking error for a 2 DOF robot with a floating base (Ulrich et al., 2012; Ulrich and Sasiadek, 2012a; Ulrich and Sasiadek, 2012b). However, this strategy leads to large motor torque values when there are sharp changes in the end-effector path. Computed torque (Yu and Chen, 2014) and feedback linearization (Nanos and Papadopoulos, 2015) have both been proposed, but both methods rely on model knowledge and are often not robust to modeling error. Singular perturbation theory has also been popular for designing flexible joint controllers, and has been applied to a dual-arm 2 DOF space robot scenario (Zarafshan and Moosavian, 2011), as well as a 2 DOF space robot with an additional flexible link (Yu and Chen, 2014). Outer-loop (i.e., link-side joint speed) control for flexible-joint manipulation of massive objects has been addressed, as well as path planning and checking planned trajectories against dynamic constraints, for fixed-base scenarios (Carabis et al., 2021). However, trajectory optimization and the influence of floating-base dynamics were not addressed in this work.

Trajectory generation for rigid space manipulators has been addressed using particle swarm optimization (Huang and Xu, 2006) and by extending the well-known minimum-time solution developed by (Bobrow et al., 1985) to floating base scenarios (Dubowsky et al., 1989). Trajectory generation for flexible-link space robot scenarios has also been addressed by (Wu et al., 2004), where oscillations during transport are minimized. However, this work does not address motor torque limits or joint flexibility, and some oscillations still persist even in simulation.

Our contribution is a model-based methodology for flexible-joint space manipulator trajectory planning. Existing control methods for flexible-joint manipulators do not account for joint speed and motor torque limits, and there will be issues with trajectory following in scenarios where these limits are reached. Our work incorporates constraints on motor torque and joint speed during the trajectory generation step, providing a methodology to plan trajectories where these constraints are satisfied. We examine both free-floating and controlled base scenarios, and incorporate base actuation (provided through reaction wheels and thrusters) constraints in the controlled base scenario. An optimization step is undertaken to reduce total trajectory time.

Furthermore, we investigate a 7 DOF space robot model with flexible joints and a free-flying base in simulation. Existing work has addressed relatively simple, low-DOF scenarios in simulation. Finally, we generate a feedforward motor signal while planning the link-side trajectory. Although disturbances and modeling errors will require feedback control on-application, we propose the feedforward motor signal as a means to further reduce oscillations during trajectory execution. We investigate this experimentally using a single flexible-joint experiment conducted on an air-bearing table, a popular method for offsetting the effects of gravity (Rybus and Seweryn, 2016).

This paper is organized as follows. Section 2 provides the problem statement and scope, Section 3 provides the problem formulation, while Section 4 details the trajectory generation algorithm and optimization problem. Sections 5 and Section 6 provide the experimental setup and results, respectively. Finally, Section 7 offers a concluding discussion and outlines potential future work. Portions of this paper have also appeared in the doctoral dissertation Carabis (2020).

## 2 Problem Statement and Scope

We assume an *n* DOF flexible-joint space robot with only rotational joints, a floating base, and a grasped load. Define $q\in R^{n}$ as the vector of link-side joint angles. Assume a desired joint-space path has been planned, which also defines the desired task-space path. Let *λ* ∈ [0, 1] be the path variable, such that *q* (*λ* = 0) defines the initial joint configuration and *q* (*λ* = 1) defines the final joint configuration. The trajectory generation step then becomes indexing the path variable with time *t*, i.e., generating *λ*(*t*).

The problem under consideration is generating the trajectory *λ*(*t*) subject to motor torque, link-side joint speed, and base actuation constraints (in the controlled base scenario). Mathematically stated, we require

$$|\dot{q}\left(t\right)|⪯{\dot{q}}_{\max},\;|\tau_{m}\left(t\right)|⪯\tau_{m,\max},\;\forall t,$$ (1)

for all scenarios considered, and

$$|f_{b}\left(t\right)|⪯f_{b,\max},\;\forall t,$$ (2)

for scenarios with a controlled base. Here, ⪯ denotes an element-wise inequality, ${\dot{q}}_{\max}$ is the link-side joint speed limit, $\tau_{m}\in R^{n}$ is the applied motor torque, *τ*_*m*, max_ is the motor torque limit, *f*_*b*_ is the spatial force applied at the base, and *f*_*b*, max_ is the base spatial force limit. Note that the spatial force is stacked torque and force, i.e., $f_{b}={\left[\begin{array}{cc} \tau_{b}^{T} & F_{b}^{T} \end{array}\right]}^{T}$ , where *τ*_*b*_ is the torque generated by thrusters or reaction wheels and *F*_*b*_ is the force generated by thrusters at the robot base.

We note that there are additional dynamic limits that may be of concern, such as the speed of the grasped object at the end effector and spatial forces at the end effector leading to slip or gripper damage. The former can be incorporated into constraints on $\dot{q}$ , while we consider the latter to be outside the scope of this work.

As there are many trajectories that would satisfy these requirements (and arbitrarily slowing a trajectory would make the constraints easier to satisfy), we propose finding a trajectory subject to the constraints while minimizing total trajectory time. Global minimum-time trajectory generation for this scenario remains an unsolved problem, and we allow for local minimum solutions. Furthermore, we allow for a sufficiently smooth trajectory shape to be assumed. Future work will seek to remove this assumption, potentially allowing for faster trajectories to be generated. Regardless, the methodology presented here provides a means for generating feasible trajectories and reducing the total trajectory time while satisfying dynamic constraints.

## 3 Problem Formulation

**FIGURE 2:**
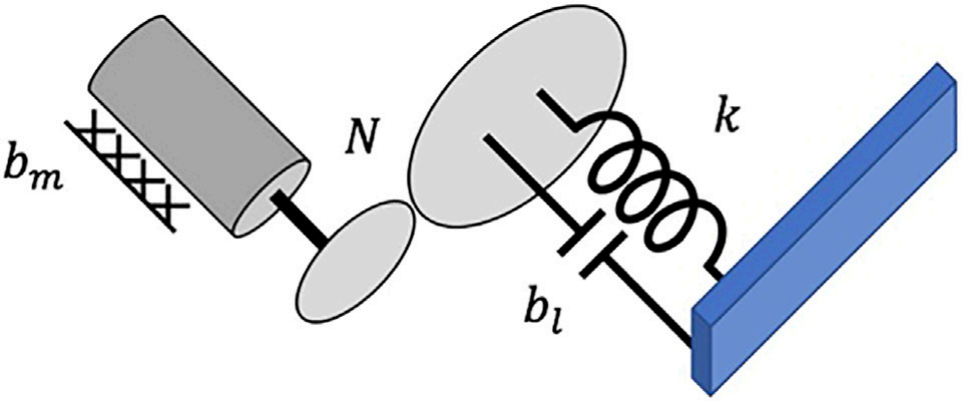
Single flexible joint. In this diagram, *b*_*m*_, *b*_*l*_, *N*, and *k* represent the motor damping, gear damping, gear ratio, and spring constant, respectively, and *i* denotes the *i*th joint.

We model the flexible joints using the same approach as (Spong, 1987). This model assumes that each flexible joint is comprised of a 1-D rotational motor inertia and a torsional spring-damper that connects the *i*th motor to the *i*th manipulator link. Furthermore, this model assumes that the motors rotate relative to an inertial frame (i.e., the inertial coupling between the links and motors is ignored). Please note that we have derived a model using the Newton-Euler approach (Jain, 2010) that includes the inertial coupling between the motors and links, but no significant error was noted in simulation. This is likely due to the large mass/inertia of the manipulator base and grasped object, and we therefore utilize the model with no inertial coupling. Figure 2 illustrates this model for a single joint.

Using the spatial algebra approach (Jain, 2010) the dynamics of the manipulator arm, floating base, and grasped object are

$$\left[\begin{array}{c} f_{b}\\ \tau \end{array}\right]=\left[\begin{array}{cc} M_{b} & M_{rb}\\ M_{br} & M_{r} \end{array}\right]\left[\begin{array}{c} \alpha_{B}\\ \ddot{q} \end{array}\right]+\left[\begin{array}{c} C_{B}\\ C_{r} \end{array}\right]+\left[\begin{array}{c} \Phi_{n+1,B}^{T}\\ J^{T} \end{array}\right]f.$$ (3)

The Supplementary Appendix provides a detailed derivation. Here, *τ* denotes the link-side torque vector. The 6 × 6 mass-inertia matrix of the robot base is given by *M*_*b*_, *M*_*r*_ is the joint-space mass-inertia matrix of the manipulator arm, *M*_*rb*_, *M*_*br*_ are the mass-inertia couplings between the manipulator base and arm, *C*_*r*_, *C*_*B*_ are the centrifugal and Coriolis terms, *J* is the well-known manipulator Jacobian, and $\Phi_{n+1,B}^{T}$ is a mapping from the external spatial force *f* (generated by the grasped object motion) to the manipulator base center-of-mass (COM) frame *B*. Note that $\Phi_{n+1,B}^{T}$ is dependent on *q*, and that *n* + 1 denotes the end effector frame. Further details on this mapping are provided in the Supplementary Appendix. The spatial acceleration of the base is given by $\alpha_{B}={\left[\begin{array}{c} {\dot{\omega }}_{B}^{T}{\dot{v}}_{B}^{T} \end{array}\right]}^{T}$ , where *ω*_*B*_ is the rotational velocity of the manipulator base frame and *v*_*B*_ is the translational velocity of the manipulator base frame.

In the case where the floating base is perfectly controlled and acts as a fixed base, Eq. 3 becomes the well-known manipulator model with joint flexibility given by (Spong, 1987). When the base is uncontrolled and free-floating, *f*_*b*_ = 0.

We assume the target object is rigidly grasped, and model the object dynamics using

$$A^{T}f=M_{c}\alpha_{c}+b_{c},$$ (4)

where *A* is the Jacobian transform between the end-effector and object COM, *M*_*c*_ is the object mass-inertia matrix (about the COM), *b*_*c*_ are the centrifugal/Coriolis terms associated with the grasped object motion, and *c* denotes the object COM frame. Since there is a single rigid grasp point, *A* ^*T*^ is invertible and we can solve for *f*. As we consider a rigid grasp scenario in this paper, the mass/inertia of the grasped payload can be combined with the end effector link (i.e., set *f* = 0 and combine mass/inertia properties of link *n* and the payload). However, the full model presented here allows for contact models to be introduced in future work, so we present the arm and payload dynamics in full.

The joint torque for each flexible joint is given by

$$\tau =K\left(N^{-1}q_{m}-q\right)+b_{l}\left(N^{-1}{\dot{q}}_{m}-\dot{q}\right),$$ (5)

where $q_{m}\in R^{n}$ is the vector of motor positions, *K* is a diagonal matrix of joint spring stiffnesses, *b*_*l*_ is a diagonal matrix of joint damping, and *N* a diagonal matrix of gear ratios. Note that we assume linear torsional springs and viscous damping. The motor dynamics are

$$I_{m}{\ddot{q}}_{m}+b_{m}{\dot{q}}_{m}+KN^{-1}\left(N^{-1}q_{m}-q\right)+b_{l}N^{-1}\left(N^{-1}{\dot{q}}_{m}-\dot{q}\right)=\tau_{m},$$ (6)

where *I*_*m*_, *b*_*m*_ are diagonal matrices of the motor inertias and viscous motor damping, respectively.

## 4 Trajectory Generation Algorithm

Our first step is to generate a feedforward motor trajectory that will result in some desired link-side trajectory, *q*_*d*_(*t*). We assume perfect motor control and consider the input to the system to be the motor trajectory, i.e. *u*(*t*) = *q*_*m*_(*t*). First, we consider a fixed-base system and assume *q*_*d*_(*t*) is provided. We combine the arm and load dynamics, as well as the portion of the joint torque dependent on link-side trajectory, into the term

$$v\left(t\right)=M_{r}\ddot{q}+C_{r}+Kq+b_{l}\dot{q}+J^{T}f.$$ (7)

Note that the load dynamics *f*(*t*) can be solved for in terms of *q*_*d*_(*t*) using velocity and acceleration propagation from the fixed-base. From Eq. 3, we can solve for *u*(*t*) = *q*_*m*_(*t*) using the low-pass filter (LPF)

$$u_{i}\left(s\right)=\frac{v_{i}\left(s\right)}{\frac{K_{i}}{N_{i}}+\frac{b_{li}}{N_{i}}s},$$ (8)

where *i* corresponds to the *i*th element of the associated diagonal matrix or vector and *s* denotes the Laplace variable. This solution is obtained by substituting Eq. 7 into Eq. 3 and solving the differential equation for *u* (*t*). For the case where we assume the base is perfectly controlled, we solve for the base spatial force by setting *α*_*B*_ = 0 and solving for *f*_*b*_ using Eq. 3. Note that the motor trajectory generation for this case is unchanged from the fixed-base scenario.

For the case where the base is uncontrolled, we must solve for *α*_*B*_(*t*) for some given *q*_*d*_(*t*) before *u*(*t*) can be computed. Using the acceleration propagation detailed in the Supplementary Appendix, we obtain

$$\alpha_{c}=\Phi_{cB}\alpha_{B}+\overset{c}{\underset{k=1}{\sum }}\Phi_{ik}\left(H_{k}{\ddot{q}}_{k}+a_{k}\right).$$ (9)

We then substitute Eq. 9 into the top portion of Eq. 3 and rearrange to solve for the base acceleration, obtaining

$$\alpha_{B}={\left(M_{B}+\Phi_{c,B}^{T}M_{c}\Phi_{c,B}\right)}^{-1}\left(-M_{rB}\ddot{q}-C_{B}-\Phi_{c,B}^{T}\left(M_{c}\overset{c}{\underset{k=1}{\sum }}\Phi_{ik}\left(H_{k}{\ddot{q}}_{k}+a_{k}\right)+b_{c}\right)\right).$$ (10)

Note that Eq. 10 is dependent on the base spatial velocity, and that to compute *α*_*B*_(*t*) at some given time we must know the base spatial velocity as a function of time, *V*_*B*_(*t*). Therefore, the base dynamics must be propagated forward in time from the initial condition to solve for *α*_*B*_(*t*). Any suitable integration method may be used to do this—for our work, we use the trapezoidal rule.

Collecting the arm, base, and grasped object dynamics together, we obtain

$$v\left(t\right)=M_{Br}\alpha_{B}+M_{r}\ddot{q}+C_{r}+Kq+b_{l}\dot{q}+J^{T}f.$$ (11)

Note that *f*(*t*) is dependent on *α*_*B*_(*t*). This signal is once again passed through Eq. 8 to obtain the correct feedforward motor trajectory for some specified *q*_*d*_(*t*).

We next propose the optimization problem to reduce the total trajectory time while satisfying dynamic constraints on joint speed, motor torque, and base spatial force (in the case of a controlled base). As previously stated, we assume the joint-space path (*q*_*d*_(*λ*)) is known and wish to index the path variable with time to generate the trajectory (i.e., find *λ*(*t*)). From Eq. 3, we find that the link-side trajectory must be sufficiently smooth to obtain finite values of *τ*_*m*_ when solving the system differential equation. Specifically, *q*(*t*) must be differentiable to the third order for systems with coupled linear damping between the motors and joints, and differentiable to the fourth order with systems with decoupled linear damping (i.e., *b*_*l*_ = 0). One such trajectory that satisfies the more stringent requirement (and therefore applies to more systems) is the bounded snap (BS) motion profile, which is similar to the commonly used bounded acceleration (trapezoidal velocity) motion profile, but with snap being a constant maximum or minimum value rather than acceleration. We use the algorithm developed by (Nguyen et al., 2008) to compute this motion profile based on the limits $\eta ={\left[\begin{array}{cccc} {\dot{\lambda }}_{\max} & {\ddot{\lambda }}_{\max} & {\overset{...}{\lambda }}_{\max} & {\overset{....}{\lambda }}_{\max} \end{array}\right]}^{T}$ and obtain *λ*(*t*). With *λ*(*t*) and *q*(*λ*) defined, we can compute *q*(*t*).

To compute the motor torque, we must have some estimate of ${\ddot{q}}_{m}$ . Our choice is to convert Eq. 6 into a discrete-time system. This does introduce some error, as we treat the motor trajectory signal as discrete time but treat the joint-side trajectory as continuous in simulation to more closely match the physical system. However, we have found that this error is generally small. Other possible choices include estimating ${\ddot{q}}_{m}$ via numerical differentiation, which would also introduce numerical error.

Consider the state-space representation of the LPF given by

$${\dot{q}}_{m}=A_{L}q_{m}+B_{L}z,$$ (12)

where $z={\left[\begin{array}{cc} q^{T} & {\dot{q}}^{T} \end{array}\right]}^{T}$ . Assuming *q*_*m*_ (0) = 0, the motor position at the *k*th step for the discrete-time system is given by

$$q_{mk}=\overset{k-1}{\underset{m=0}{\sum }}A_{Ld}^{k-m-1}B_{Ld}z_{k},$$ (13)

where *A*_*Ld*_, *B*_*Ld*_ correspond to the discrete-time versions of *A*_*L*_, *B*_*L*_, respectively. From Eq. 6, we write the state-space representation of the motor dynamics as

$$\dot{x}=Ax+B\tau_{m}+B_{0}z,$$ (14)

$$q_{m}=Cx,$$ (15)

where $x={\left[\begin{array}{cc} q_{m}^{T} & {\dot{q}}_{m}^{T} \end{array}\right]}^{T}$ and $C=\left[\begin{array}{cc} I & 0 \end{array}\right]$ , with **I** denoting the identity matrix. Once again assuming *q*_*m*_ (0) = 0, the discrete time system provides

$$q_{mk}=\overset{k-1}{\underset{m=0}{\sum }}CA_{d}^{k-m-1}B_{d}\tau_{m}+\overset{k-1}{\underset{m=0}{\sum }}CA_{d}^{k-m-1}B_{0d}z,$$ (16)

where *A*_*d*_, *B*_*d*_, *B*_0*d*_ correspond to the discrete-time versions of *A*, *B*, and *B*_0_, respectively.

Let ${\bar{\tau }}_{m}$ , $\bar{V}$ , and $\bar{z}$ be the stacked vectors of each time-step *k* covering the entire trajectory of *τ*_*m*_(*t*), *v*(*t*), and *z*(*t*), respectively. Rearranging Eq. 13, and Eq. 16 in matrix form for the entire trajectory, we obtain

$$L\bar{V}=H{\bar{\tau }}_{m}+P\bar{z},$$ (17)

$${\bar{\tau }}_{m}=H^{-1}\left(L\bar{V}-P\bar{z}\right),$$ (18)

where *L* maps the signal *v*(*t*) to the motor position through the LPF, *H* maps the applied motor torque to the motor position, and *P* maps the joint-side trajectory to the motor position. This provides a way to solve for the motor torque over the entire trajectory.

Finally, we pose the optimization problem

$$\begin{array}{ll} & \underset{\eta }{\min}T_{f}\left(\eta \right)\\ \text{s.t.}\;|\tau_{m}|⪯\tau_{\max},\; & |\dot{q}|⪯{\dot{q}}_{\max},\;|f_{b}|⪯f_{\max}, \end{array}$$ (19)

where *T*_*f*_(*η*) denotes the final time of the trajectory. We note that *T*_*f*_(*η*) is obtained via a numerical algorithm in (Nguyen et al., 2008), so we omit the computation of *T*_*f*_(*η*) here for brevity and direct the reader to the citation. We propose solving this optimization problem using an iterative constrained nonlinear optimization method, such as sequential quadratic programming (SQP).

## 5 Experimental Setup

### 5.1 Experimental Setup: Computer Simulation

We choose the Rethink Robotics Baxter robot (Rethink Robotics, 2018) as a stand-in for a space manipulator in our simulation. This robot has 7 DOF and series-elastic actuators, meaning that it exhibits significant joint-flexibility. The link mass and inertia, link COM, and joint spring coefficients are all available from (Rethink Robotics, 2018). We choose the computer simulation motor inertias, motor damping, joint damping, and gear ratios as shown in Table 1.

**TABLE 1:** Assumed Baxter robot parameters.

Joint	*I*_*mi*_ (kg m^2^)	*b*_*mi*_ (kg m^2^/sec)	*N*_*i*_
1	0.5E−3.5	8	20
2	0.5E−3.5	8	20
3	0.5E−3.5	8	20
4	0.5E−3.5	8	20
5	0.5E−3.5	6	18
6	0.5E−3.5	6	18
7	0.5E−3.5	6	18

**FIGURE 3:**
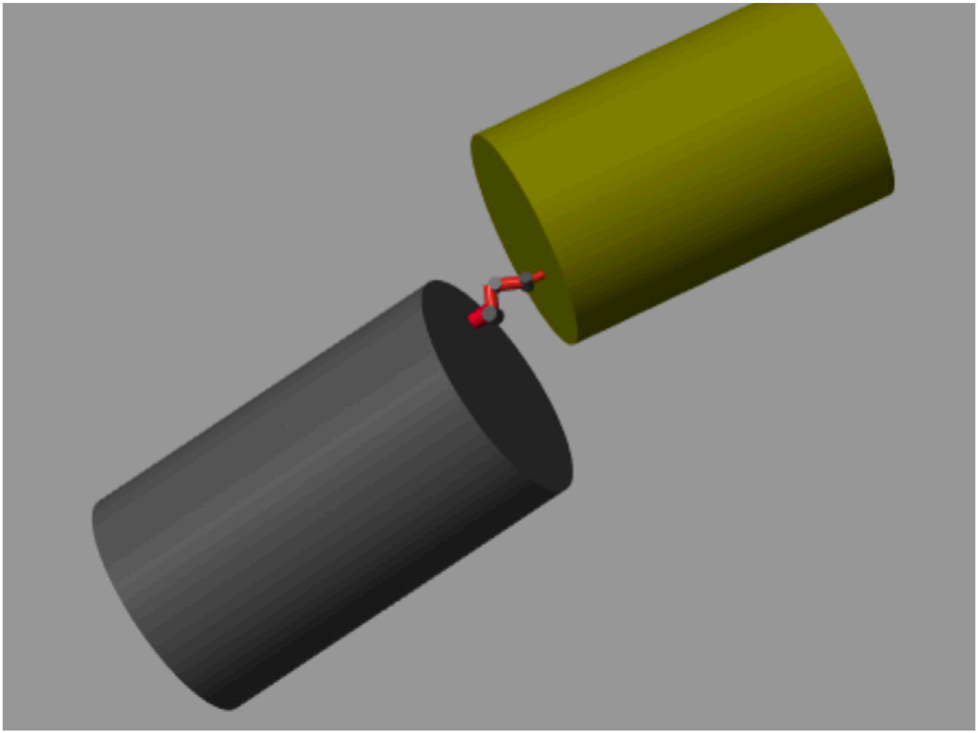
Simulation model of Baxter arm with target satellite (gold) and base satellite (gray).

We choose the satellite parameters available at (Kramer, 2002), and model both the target and base satellites as cylinders with evenly distributed mass, with parameters provided in Table 2. Base parameters are chosen at a similar scale, but slightly larger, with parameters provided in Table 2. Both the robot base and grasp point are located on the flat surfaces of the cylindrical satellites, as shown in Figure 3. There is a 1 m offset from the center axis of the base satellite to the first joint of the robot arm and a 0.5 m offset from the center axis of the target satellite to the grasp point.

**TABLE 2:** Assumed base and target satellite parameters.

Satellite	mass (kg)	radius (m)	length (m)
Target	2,200	0.7	4.3
Base	6,600	1.5	5

Full spatial simulations are conducted with MATLAB and the Simscape Multibody package. A separate dynamic simulation that employs the Newton-Euler algorithm to solve the equations of motion is also implemented using MATLAB and Simulink. The results of these simulations have been compared to confirm the Multibody simulation results match the dynamics shown in Eq. 3. The optimization problem is solved using the SQP algorithm with the built-in MATLAB *fmincon* function.

### 5.2 Experimental Setup: Physical Experiment

**FIGURE 4:**
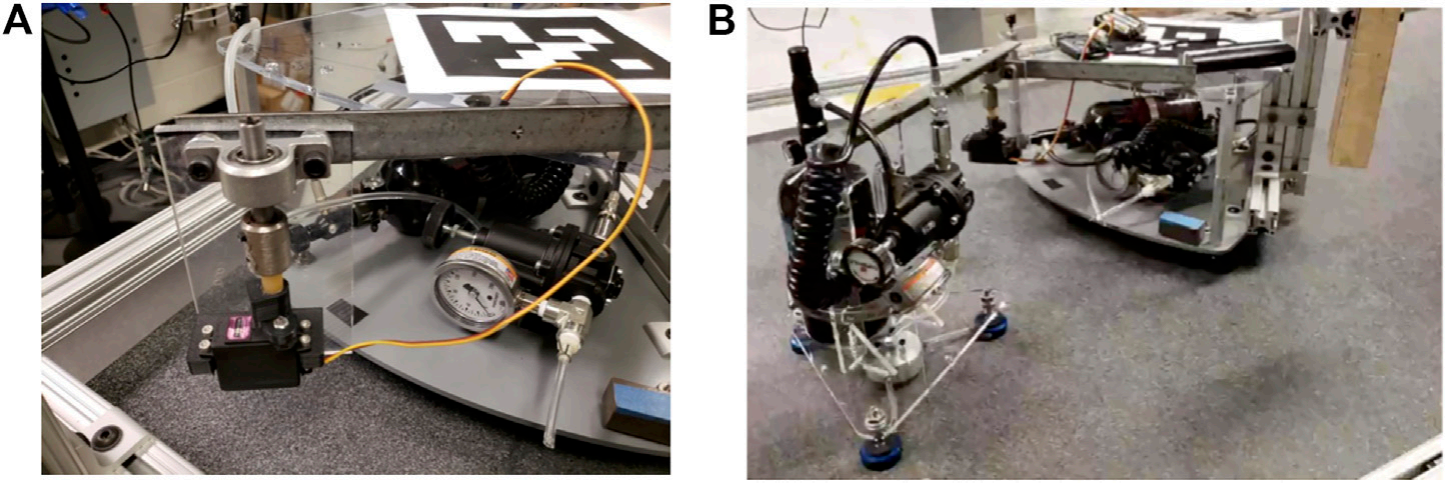
Flexible joint experimental setup: **(A)** detailed view of custom flexible joint, **(B)** air bearing bodies connected via flexible joint.

To investigate the efficacy of the feedforward motor trajectory for oscillation reduction, we construct a single flexible joint for our physical experiment. The joint is composed of a TowerPro MG995R servo motor, a 25.4 mm length section of ∼9 mm diameter 60 A durometer polyurethane, and a rigid appendage connected to a second air bearing object, as shown in Figure 4. We mount this joint to two air bearing objects, which use three 40 mm diameter Newway air bearings each to reduce friction between the objects and a Mojave granite table. On-board air is supplied using compressed gas cylinders, as shown in Figure 4. The air supply to the base object is turned off during our experiments, making the system a fixed-base 1 DOF robot. The floating air bearing object has a mass of approximately 4.1 kg.

We use two fiducial tags, one mounted on each air bearing object, to estimate the link-side joint position. Computer vision data is taken using a 1.3 mega-pixel FLIR color Flea3 machine vision camera at approximately 20 Hz. The servo motor is controlled via a Raspberry Pi 3 with a servo control hat. We assume that the servo motor control loop is tight, and therefore that the commanded servo motor position is equivalent to the actual servo motor position. The Raspberry Pi code and computer vision code are integrated using Robot Raconteur (RR) (Wason, 2016) at approximately 20 Hz so that time-stamped vision and motor data can be recorded. We slow the command/sample rate using the same strategy as (Peng et al., 2018) to achieve a consistent 20 Hz.

## 6 Experimental Results

### 6.1 Simulation Experiment Results

First, we check the efficacy of the feedforward motor trajectory given by Eq. 8 to remove oscillations in simulation. As our model knowledge is complete, all oscillations should be successfully removed. However, modeling error and disturbances will require additional feedback control in a real physical system. We investigate the feedforward motor trajectory in part to ensure our derivations and algorithms for generating *u*(*t*) are correct. Furthermore, we compare the feedforward motor trajectory to the case where rigid joints are assumed (denoted the naïve case; *u*(*t*) = *Nq*_*d*_(*t*)). Note that the naïve method suffices when joints are sufficiently rigid, and tracking is perfect in the infinitely stiff (rigid) joint case. This comparison shows the significance of the flexible joint dynamics in the space robot application, and what issues may arise if rigid joints are assumed during trajectory generation or trajectory following.

We first compare simulation results for a fixed-base scenario. A desired link-side BS trajectory is generated for this test case using $\eta ={\left[\begin{array}{cccc} 0.04 & 0.05 & 0.02 & 0.3 \end{array}\right]}^{T}$ , with a final time of approximately 27.9 s. We use a previously generated path, *q*_*d*_(*λ*), for all cases considered, which involves 6-DOF motion of the target object and motion of all of the 7 robot joints.

**FIGURE 5:**
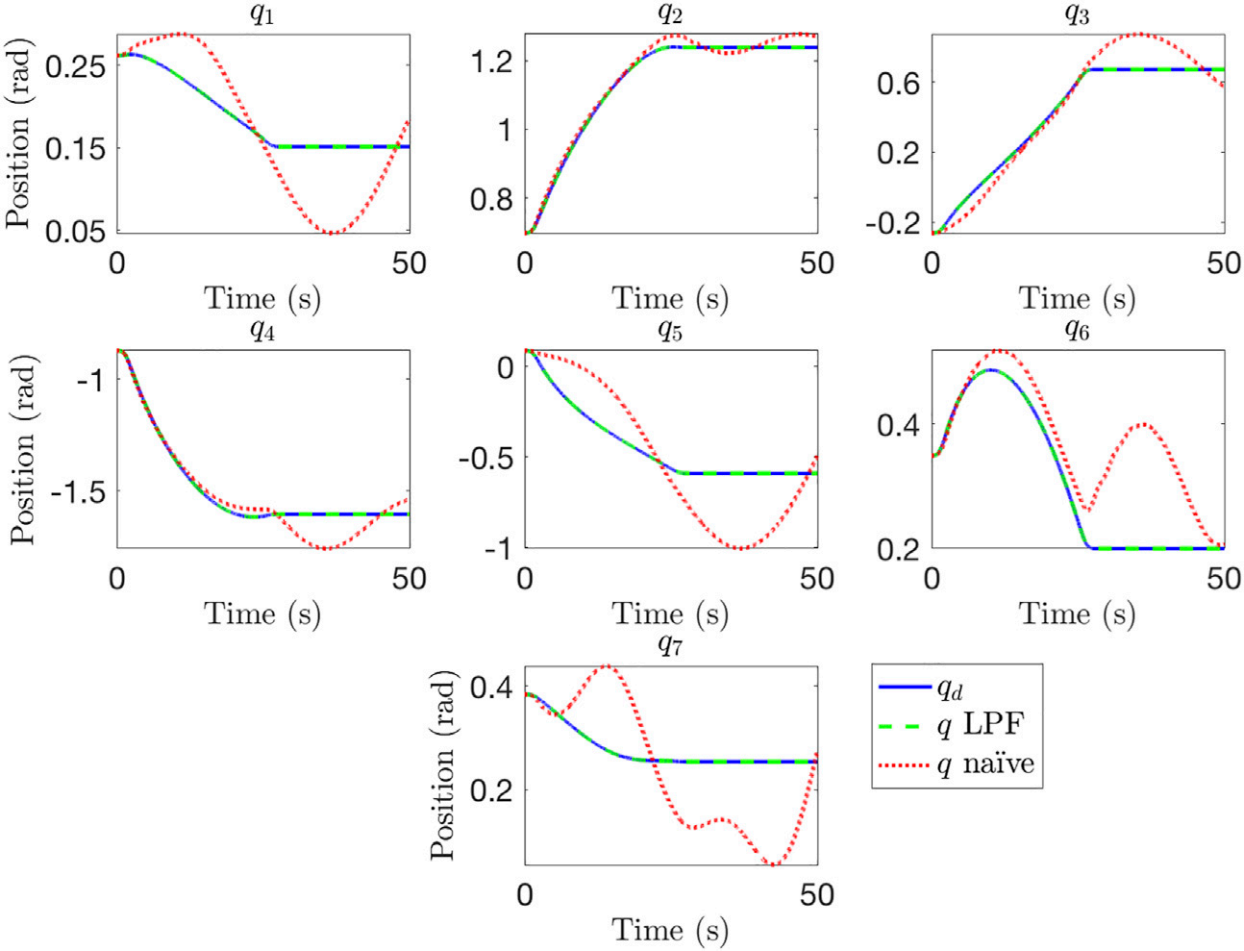
Comparison of joint space performance for a naïve motor trajectory and a trajectory generated using the LPF method.

The joint-space performance results are provided in Figure 5. It is clear from these results that the naïve motor trajectory is not sufficient, with oscillations continuing well after the trajectory has been completed (at ∼30 s), even in cases where position tracking had decent accuracy during desired motion, such as joints 2, 3, 4, and 6. These oscillations are particularly dangerous, as they correspond to link-side motion after the motors have reached steady-state. Physically, this is analogous to a “brakes-on” condition, meaning that simply applying motor brakes at the end of the naïve motor trajectory will lead to significant oscillations and motion that may damage the robot arm or lead to collisions between the satellites. In comparison, the motor trajectory generated using the LPF approach has good joint-space performance. We do note that choosing a large zero-order hold (ZOH) step size for the motor trajectory can lead to oscillations even when using the LPF approach, especially for faster trajectories. This should be a consideration for physical implementation of this algorithm. Comparison results are presented for a 400 Hz motor control loop.

**FIGURE 6:**
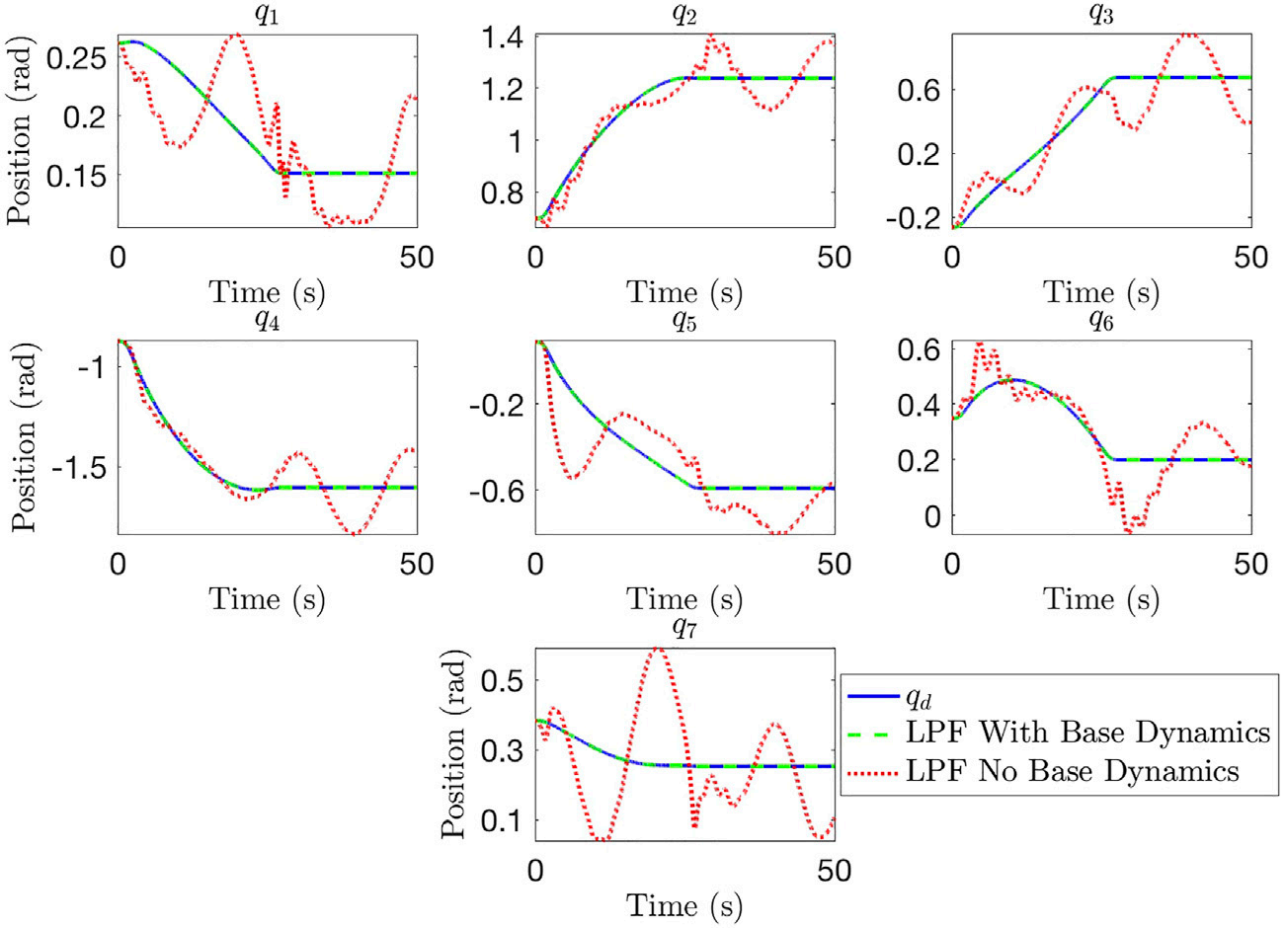
Comparison of joint space performance with uncontrolled floating base when base dynamics are and are not accounted for.

Next, we consider the importance of accounting for the floating base dynamics. Once again, we investigate this scenario in part to confirm our derivations and algorithms for generating *u*(*t*). In cases where the robot base satellite is far more massive than the target satellite, and the robot arm itself, the base dynamics can be ignored and the robot can be treated as a fixed-base manipulator. However, in cases where the base is not sufficiently massive the base dynamics can have a large impact on the motion of the manipulator arm and captured satellite, as we will show. We compare motor trajectory generation without accounting for the base dynamics (using Eq. 7, and Eq. 8 against trajectory generation accounting for the base dynamics (using Eq. 11, and Eq. 8 in Figure 6. The BS trajectory limits used in the fixed-base example are used again here. These results show that failure to account for the uncontrolled base dynamics can result in poor tracking performance and oscillations, even when using the LPF trajectory generation approach. Accounting for the base dynamics leads to good tracking performance, showing the importance of taking base dynamics into account when joint flexibility and a payload of comparable mass to the base are both present.

**FIGURE 7:**
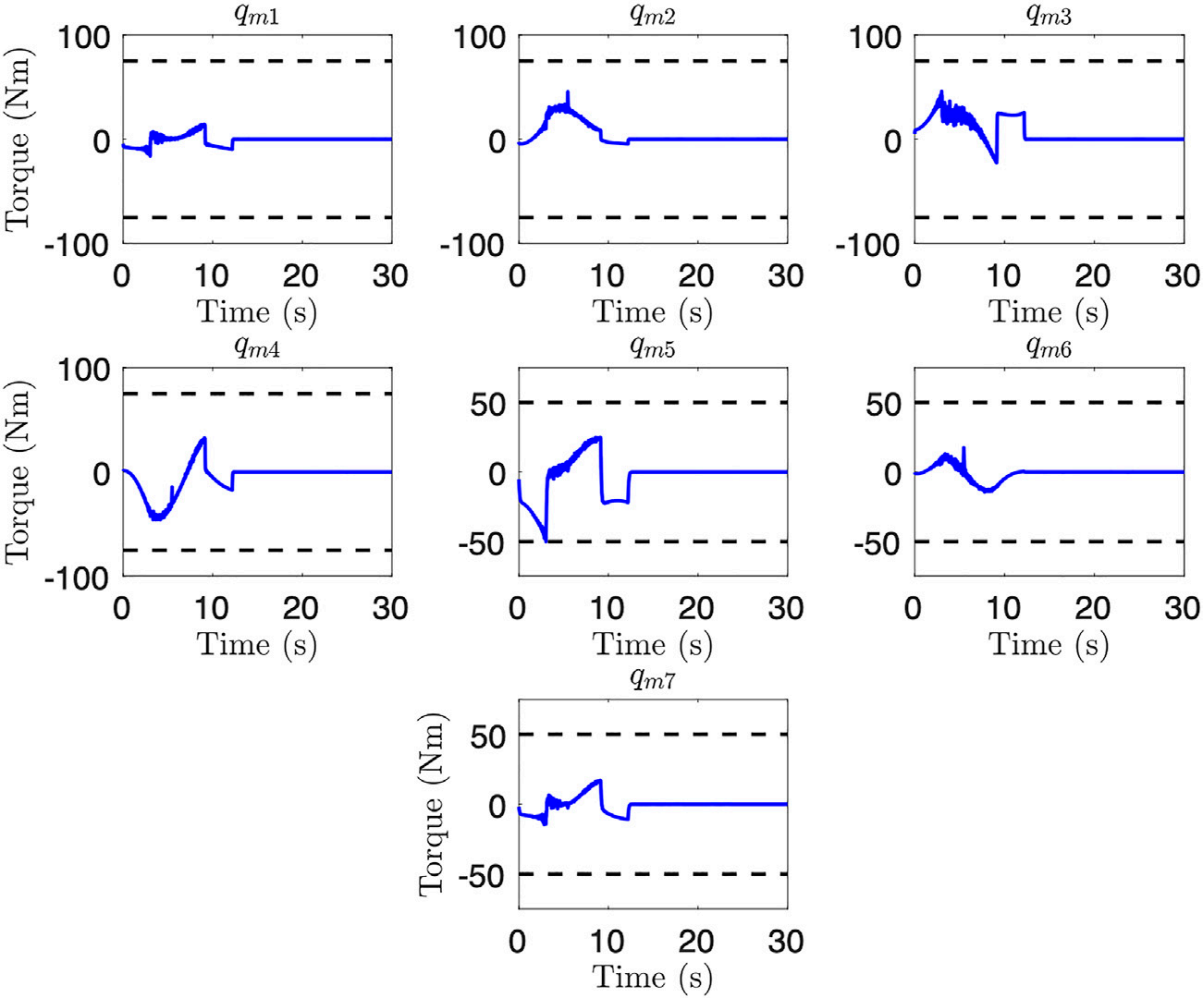
Simulated motor torques for the uncontrolled floating base trajectory after optimization. Note that joint 5 reaches the lower torque limit.

With a methodology for generating *u*(*t*) = *q*_*m*_(*t*) given some *q*_*d*_(*t*), the next question is how to generate *q*_*d*_(*t*) itself subject to the constraints previously outlined. We utilize the *fmincon* function within MATLAB to implement Eq. 19 with an SQP algorithm. An uncontrolled floating base is assumed for these trajectory generation scenarios. We slow the assumed motor control loop to 50 Hz to reduce the computational requirement for checking dynamic constraints, with small loss of accuracy in the computation of motor torque and base actuation. Furthermore, we choose $\tau_{\max}={\left[\begin{array}{ccccccc} 75 & 75 & 75 & 75 & 50 & 50 & 50 \end{array}\right]}^{T}$ Nm. The BS trajectory limits used in the previous example are treated as the initial guess, *η*_0_. Figure 7 presents motor torque results for an optimized trajectory obtained for an uncontrolled floating base scenario. As these results show, joint 5 reaches the torque limit. A final trajectory time of 12.2 s was obtained, which is a 56% improvement over the initial guess, and illustrates the benefits of undertaking the optimization step.

**FIGURE 8:**
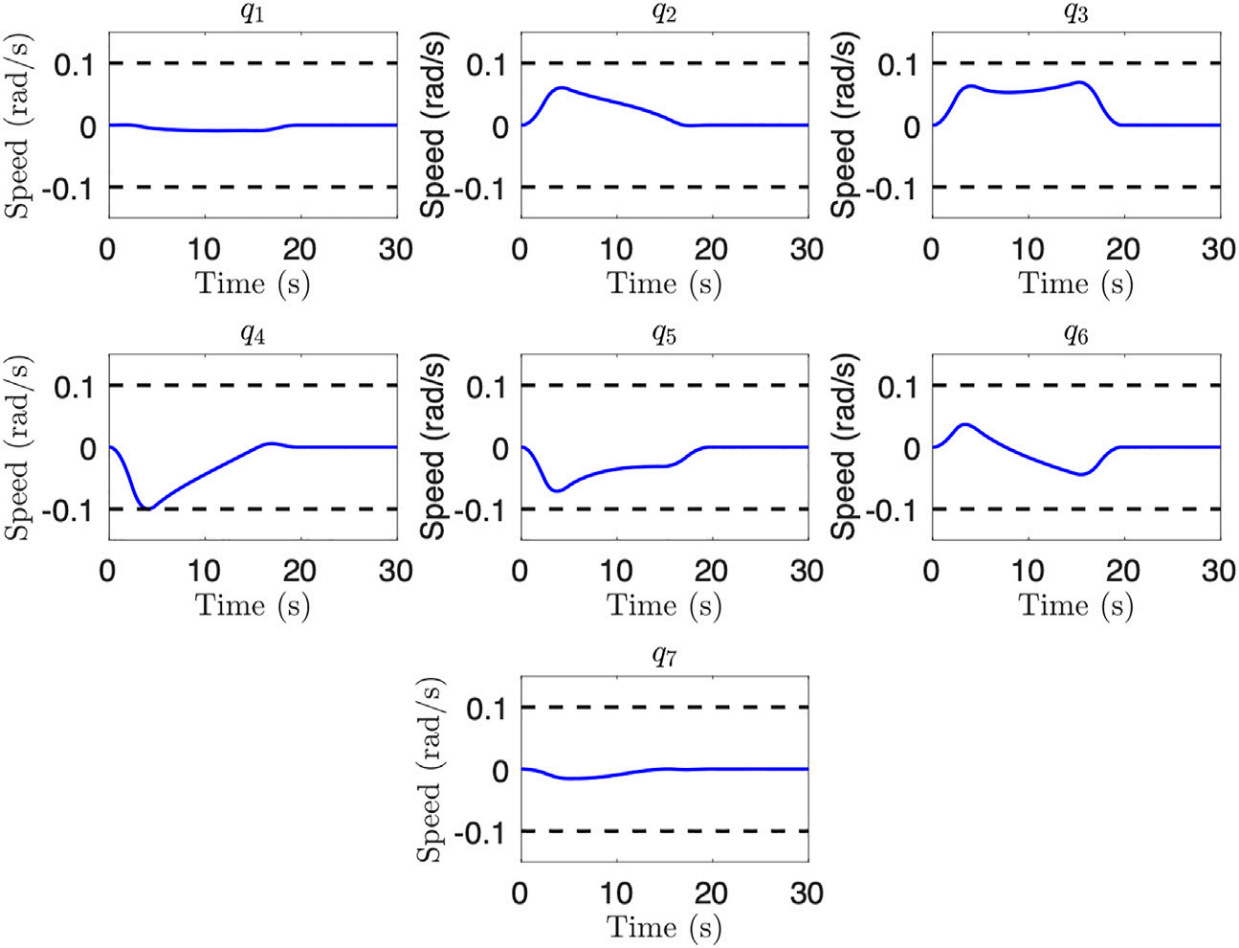
Joint speeds for an uncontrolled floating base scenario after optimization where joint speed is the limiting factor. Note that joint 4 reaches the lower joint speed limit.

We also examine a case with link-side joint speed limits, and add the constraint ${\dot{q}}_{\max}=0.1$ rad/s for each joint to the previously considered case. Figure 8 shows the joint speed limits for this case. We see that joint 4 reaches the joint speed limit, showing how different joints may be the limiting factor for the same path depending on the dynamic constraints. A final trajectory time of 19.7 s, a 29% improvement over the initial guess, was obtained. These results show the efficacy of the trajectory generation for reducing total trajectory time subject to dynamic constraints.

**FIGURE 9:**
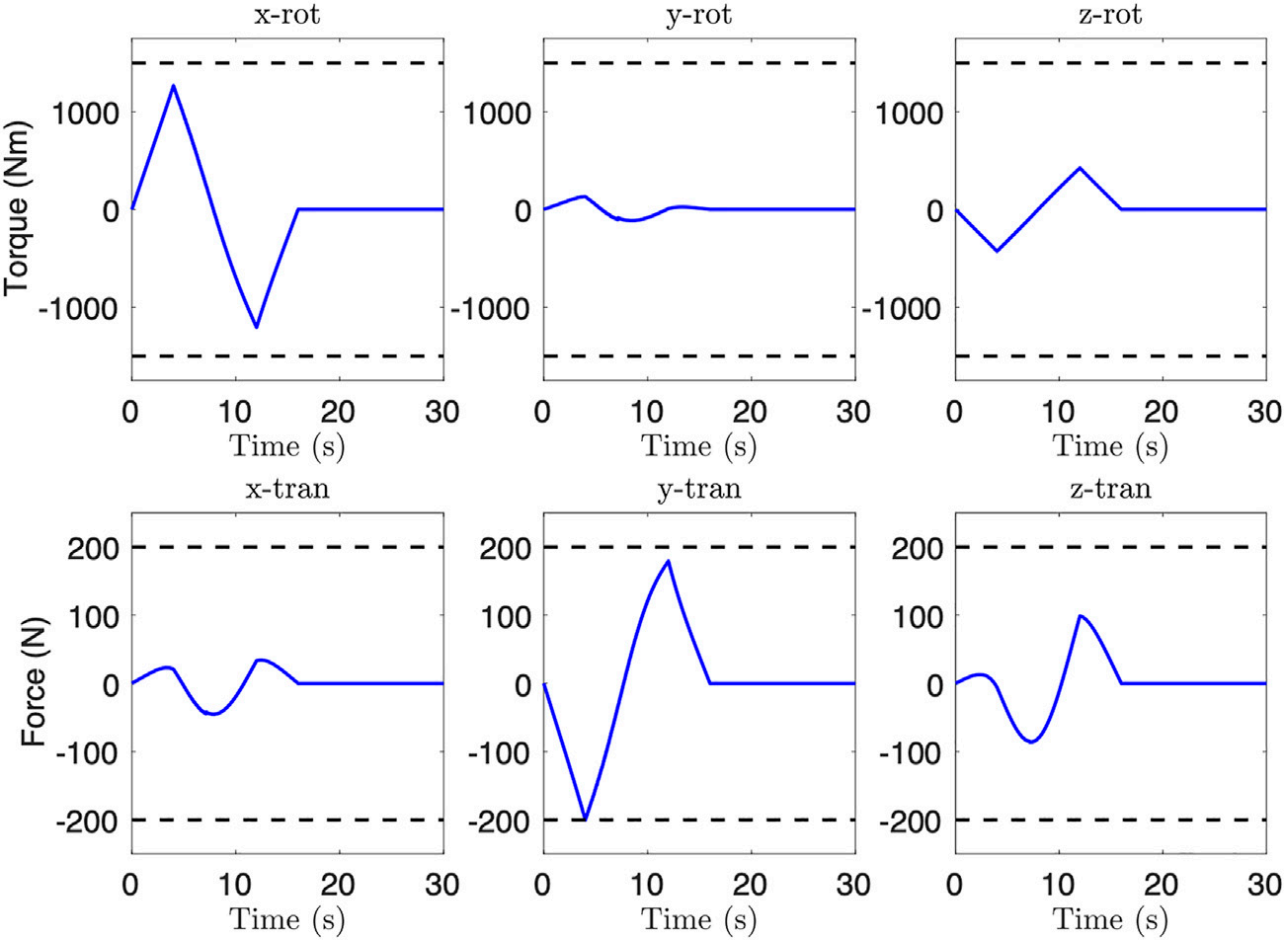
Feedforward base actuation during controlled base trajectory after optimization. Note that the *y*-direction force reaches the specified limit.

Finally, we consider the case with a controlled floating base, and choose *f*_max_ = (1,500, 200) Nm, N for base actuation torque and force limits, respectively. Figure 9 shows the base actuation required to keep the base stationary during the generated trajectory, where the *y* direction force hits the specified limit. The final trajectory time is 16.0 s, which is a 43% improvement.

It is important to note that feedback control will be needed on each joint and on the base actuators (if controlled) on-application. Therefore, the actual joint torque and base actuation will likely be higher. It is therefore important to include a safety margin when specifying dynamic limits. One method is simply to set the safety margin as a percentage of the total limit, e.g., setting a 50% margin on a motor saturation of 5 Nm would generate a torque limit for trajectory generation of 2.5 Nm.

### 6.2 Physical Experiment Results

We include the physical experiments to show the efficacy of providing a feedforward motor trajectory to reduce oscillations during trajectory following. This feedforward trajectory can be used in tandem with one of the flexible joint control options described in the Introduction to further reduce oscillations. We first perform model identification on our physical testbed by finding the frequency response to a known Schroeder-phase input signal (Bayard, 1993). Based on the physical model of a single flexible joint (Figure 2), we fit the frequency response to a linear transfer function with the following two constraints: zeros must be in the left half plane, and the DC gain is 1. We obtain the linear transfer function *G*(*s*) from *q*_*m*_(*s*) to *q*(*s*) given by

$$G\left(s\right)=\frac{q\left(s\right)}{q_{m}\left(s\right)}=\frac{0.0695}{0.281s^{2}+0.0\;322s+0.0695}.$$ (20)

**FIGURE 10:**
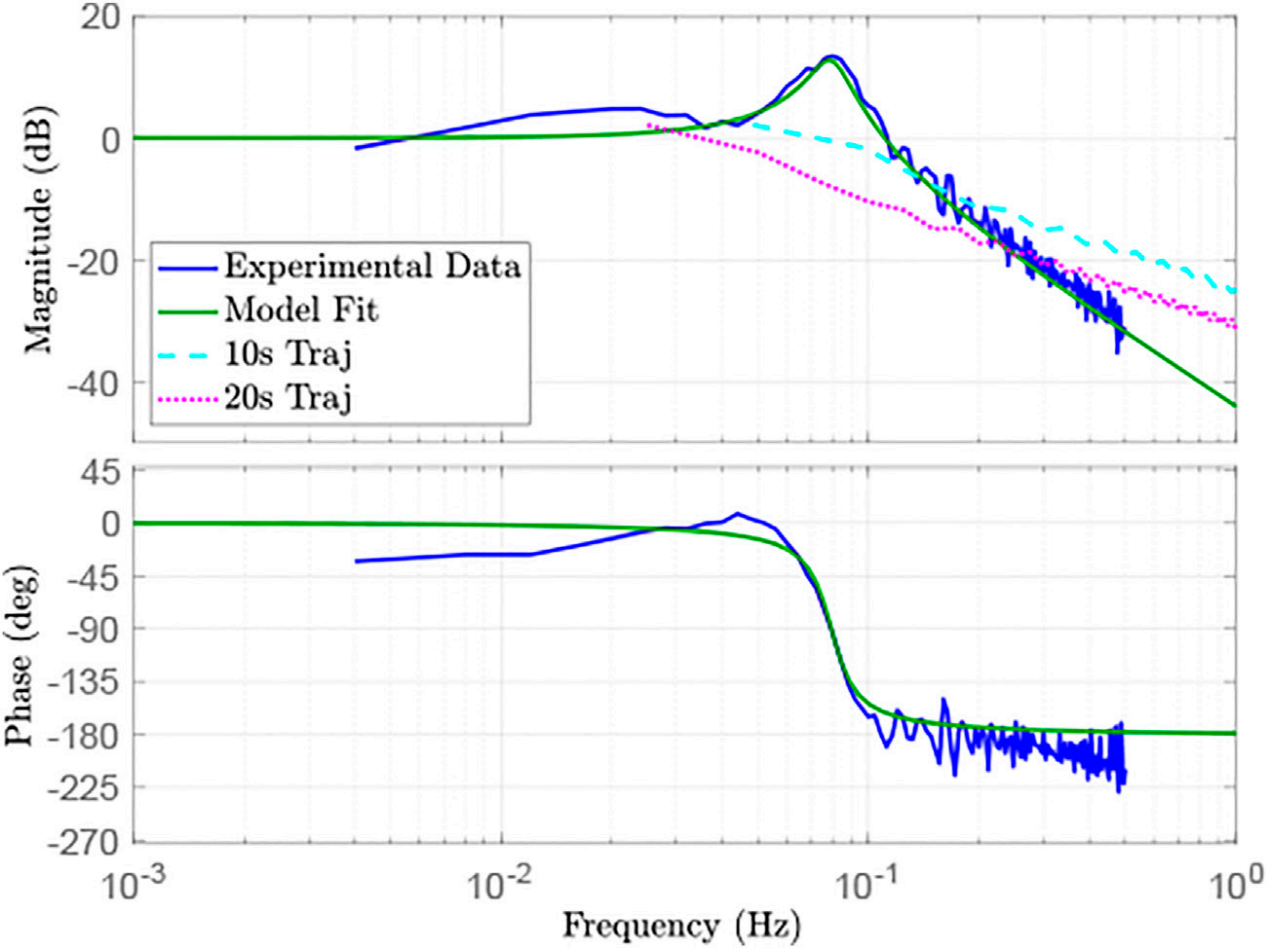
Frequency response of model fit, experimental data, and motor trajectories.

We note that this transfer function fits a model with negligible coupled damping between the motor and link sides (i.e., *b*_*l*_ = 0). This corresponds with little damping in the flexible transmission of the physical system. Figure 10 shows the frequency response of *G*(*s*) compared against the experimental data, showing the accuracy of the model fit.

We employ a similar methodology as detailed above to generate the feedforward motor trajectory, and invert *G*(*s*) to generate *q*_*m*_(*t*) given *q*_*d*_(*t*) (i.e., solving the differential equation for *q*_*m*_(*t*)). Note that inversion is possible even though *G*(*s*) is a proper transfer function because *q*_*d*_(*t*) is known *∀t*.

**FIGURE 11:**
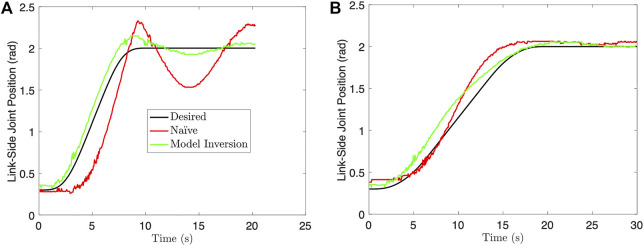
Single joint experimental results: **(A)** 10 s trajectory, **(B)** 20 s trajectory.

We investigate two test cases and repeat all experiments 5 times: a 10 s BS and 20 s BS motion profile for *q*_*d*_(*t*). Figure 11 provides representative results for both of these cases, while Table 3 provides the mean root-mean-squared-error (RMSE) and standard deviation (STD) of the RMSE over all trials. From these results, we see a clear reduction in oscillations and better tracking performance for the 10 s trajectory case. Furthermore, all 5 trials using the naïve choice (*q*_*m*_(*t*) = *q*_*d*_(*t*)) for the 10 s trajectory resulted in collision between the two air bearing objects. No collisions occurred when using the model inversion approach.

**TABLE 3:** Mean RMSE and STD of trajectory error.

Trajectory	10s Naïve	10s Model Inv.	20s Naïve	20s Model Inv.
RMSE (rad)	0.308	0.242	0.107	0.082
STD	0.03	0.02	0.01	0.003

However, we do not see significant improvement for the 20 s trajectory case. Figure 10 shows the magnitude response of the desired link-side trajectory for both trajectory cases. This result suggests that the 20 s trajectory case does not excite the flexible mode at ∼0.075 Hz, whereas the 10 s trajectory case does. Therefore, there is little benefit to using the feedforward motor trajectory for oscillation reduction in the 20 s case. This also suggests that slowing the trajectory may be sufficient to avoid oscillations with joint flexibility and the naïve feedforward approach, at the cost of total trajectory time. Finally, the feedforward motor trajectory is not enough to completely remove oscillations, and the tracking performance can still be improved. This is to be expected due to modeling error, unmodeled dynamics, and environmental disturbances. Future work will investigate using feedback control on top of the presented feedforward methodology to further reduce oscillations and improve tracking performance.

## 7 Conclusion and Future Work

This paper provides a model-based methodology for trajectory generation for a flexible-joint space manipulator grasping a massive object. We assume that the joint-space path has already been generated, and that trajectory generation indexes the path variable with time subject to dynamic constraints on motor torque, joint speed, and floating base actuation. Furthermore, we consider cases with both an uncontrolled floating base and a controlled floating base. Methodologies are provided to generate a feedforward motor trajectory given a desired link-side trajectory, and to generate a desired link-side trajectory while minimizing total trajectory time subject to dynamic constraints.

Full-spatial simulation results show the efficacy of this trajectory generation method. We also present physical experiment results for a single-joint scenario, showing that the feedforward motor trajectory can be used to improve tracking performance and oscillation reduction for certain trajectories in physical systems as well. However, some oscillations and tracking error remain, as expected. The addition of existing feedback controllers for flexible joint systems would likely further reduce these oscillations. The results also suggest that flexible modes may be avoided by using slower trajectories, albeit at the cost of trajectory time.

Future work will incorporate feedback control for further vibration suppression and tracking performance improvement. Motor control dynamics can also be considered for higher fidelity trajectory generation. Furthermore, future work will investigate more complex scenarios on the air bearing table. These scenarios will include controlled and uncontrolled floating bases, as well as higher DOF robots. We also note the assumption of a pre-planned path may impose limitations on trajectory optimizaiton. In particular, redundancy resolution cannot be leveraged during optimization, and certain paths may make satisfying dynamic constraints more difficult (e.g., large path curvature at a weaker wrist joint). These concerns should be addressed by allowing the joint-space path to be iteratively modified. Future work will investigate these improvements to the trajectory generation methodology, as well as incorporate practical kinematic path generation concerns like obstacle avoidance, joint limit constraints, and singularity avoidance.

The trajectory parameterization choice may also limit optimization. We have chosen a small number of optimization variables to parameterize the trajectory (a total of four), but this may result in certain portions of the trajectory being artificially slowed. A small portion of the path (e.g., one with high curvature with respect to the path variable) may also limit the optimized trajectory by reaching dynamic constraints at lower velocities and accelerations than other portions of the path would reach constraints at. Future work should investigate other trajectory parameterizations, such as polynomial or piece-wise definitions, which can allow for more complex trajectory shapes and for portions of the path to be optimized separately. We do note that computation time is a practical concern, so any parameterization choice with additional complexity should be evaluated for speed of computation as well. Piece-wise definitions of the trajectory should also take care to not introduce discontinuities or high acceleration, jerk, or snap in transition regions, as these could lead to oscillatory behavior or difficulties in satisfying dynamic constraints.

## Data Availability

The raw data supporting the conclusion of this article will be made available by the authors, without undue reservation.
