# Supplementary material for: Trajectory Generation for Flexible-Joint Space Manipulators
Source: Front Robot AI. 2022 Mar 31;9:687595. doi: 10.3389/frobt.2022.687595 (PMC9009532; doi:10.3389/frobt.2022.687595)
Supplement: Supplementary file 1 [file DataSheet1.pdf]

APPENDIX A: MODEL DERIVATION

This Appendix details the derivation of the flexible-joint space manipulator with floating base model. The flexible joint model is based on (Spong, 1987), while the spatial algebra approach (utilizing the Newton-Euler method) used to derive the arm dynamics is based on (Jain, 2010).

We make two key assumptions in our derivation: that the motors can be modeled by 1-D rotational inertias, and that the kinetic energy of the motors is due only to their own rotation relative to the stator (i.e., we ignore the inertial coupling between the motors and the arm links). The motor dynamics are then easily derived through applying Euler's laws, and are presented in Section 3.Similarly, the joint torque τ due to the flexible connection between the motor and link is easily derived and presented in Section 3.

We will consider a serial chain with only rotation joints, with $i = B, 1, ...n$. Let h_i, f_i be the axis of rotation of the ith joint and the spatial force at the ith joint, represented in the ith joint frame. Define

$$H_i \in \mathbb{R}^6 = \begin{bmatrix} h_i \\ 0 \end{bmatrix}, \; \forall i = 1, ...n. \tag{A.1}$$

Therefore,

$$\tau_i = H_i^T f_i. \tag{A.2}$$

Note that $H_B = \mathbf{I} \in \mathbb{R}^{\mathbf{6}}$, where $\mathbf{I}$ is the identity matrix.

We first consider the boundary conditions. Assuming negligible gravitational effects, we set the boundary conditions as

$$V_0 = 0, \quad \alpha_0 = 0, \quad f_{n+1} = f, \tag{A.3}$$

where V_0 and α_0 are the spatial velocity and acceleration boundary conditions, respectively. Here, $n + 1$ denotes the end effector frame.

Next, we consider the spatial velocity and acceleration propagation. The spatial velocity propagation is given by

$$V_{i+1} = \Phi_{i+1,i} V_i + H_{i+1} \dot{q}_{i+1}, \tag{A.4}$$

where

$$\Phi_{i+1,i} = \begin{bmatrix} R_{i+1,i} & 0 \\ -R_{i+1,i} p_{i,i+1}^{\times} & R_{i+1,i} \end{bmatrix}. \tag{A.5}$$

Here, R_{ij} denotes the rotation matrix from the ith frame to the jth frame, and p_{ij} denotes the vector from the ith frame to the jth frame, represented in the ith frame. Note that $\Phi_{i+1,i}$ is therefore dependent on q. The spatial acceleration propagation is given by

$$\alpha_{i+1} = \Phi_{i+1,i} \alpha_i + H_{i+1} \ddot{q}_{i+1} + a_{i+1}, \tag{A.6}$$

where

$$a_{i+1} = \begin{bmatrix} (R_{i+1,i} \omega_i)^{\times} \omega_{i+1} \\ R_{i+1,i} \omega_i^{\times} \omega_i^{\times} p_{i,i+1} \end{bmatrix}, \tag{A.7}$$

for robots with all rotational joints. The rotational velocity of the ith frame is given by ω_i. Note that $a_B = 0$.

The Newton-Euler equations for the ith body provide

$$M_i \alpha_i + b_i = f_i - \Phi_{i+1,i}^T f_{i+1}, \tag{A.8}$$

where

$$M_i = \begin{bmatrix} (I_i)_i & m_i p_{i,c}^\times \\ -m_i p_{i,c}^\times & m_i \mathbf{I} \end{bmatrix}, \tag{A.9}$$

and

$$b_i = \begin{bmatrix} \omega_i^\times (I_i)_i \omega_i \\ m_i \omega_i^\times \omega_i^\times p_{i,c} \end{bmatrix}. \tag{A.10}$$

The inertia matrix of the ith link at and represented in the ith coordinate frame is given by $(I_i)_i$, while the mass of the ith link is denoted by m_i. The vector from the ith frame to the ith link COM, represented in the ith frame, is given by p_{ic}.

We can then generalize these equations, noting that the spatial acceleration boundary condition is 0, and obtain

$$\alpha_i = \sum_{k=B}^{i} \Phi_{ik}(H_k \ddot{q}_k + a_k), \tag{A.11}$$

and

$$f_i = \Phi_{n+1,i}^T f + \sum_{k=i}^{n} \Phi_{ki}^T (M_k \alpha_k + b_k). \tag{A.12}$$

Define the stacked vectors and block matrices

$$\alpha = \begin{bmatrix} \alpha_1 \\ \vdots \\ \alpha_n \end{bmatrix}, \quad \bar{f} = \begin{bmatrix} f_1 \\ \vdots \\ f_n \end{bmatrix}, \quad a = \begin{bmatrix} a_1 \\ \vdots \\ a_n \end{bmatrix},$$

$$b = \begin{bmatrix} b_1 \\ \vdots \\ b_n \end{bmatrix}, \quad B = \begin{bmatrix} 0 \\ \vdots \\ 0 \\ \Phi_{n+1,n}^T \end{bmatrix},$$

$$H = \begin{bmatrix} H_1 & & 0 \\ & \ddots & \\ 0 & & H_n \end{bmatrix}, \quad M = \begin{bmatrix} M_1 & & 0 \\ & \ddots & \\ 0 & & M_n \end{bmatrix},$$

and

$$\Phi = \begin{bmatrix} \Phi_{11} & & 0 \\ \vdots & \ddots & \\ \Phi_{n1} & \dots & \Phi_{nn} \end{bmatrix}.$$

These vectors and matrices are used to derive the standard manipulator arm (fixed base) dynamics. We include them to show the relationship between the floating base and fixed base models.

Including the base, define the block matrices

$$\alpha^* = \begin{bmatrix} \alpha_B \\ \alpha \end{bmatrix}, \quad f^* = \begin{bmatrix} f_B \\ \bar{f} \end{bmatrix}, \quad a^* = \begin{bmatrix} 0 \\ a \end{bmatrix},$$

$$b^* = \begin{bmatrix} b_B \\ b \end{bmatrix}, \quad B^* = \begin{bmatrix} 0 \\ B \end{bmatrix},$$

$$H^* = \begin{bmatrix} H_B & 0 \\ 0 & H \end{bmatrix}, \quad M^* = \begin{bmatrix} M_B & 0 \\ 0 & M \end{bmatrix},$$

and

$$\Phi^* = \begin{bmatrix} \Phi_{BB} & \dots & 0 \\ \vdots & & \Phi \\ \Phi_{nB} & & \end{bmatrix}.$$

Therefore,

$$\alpha^* = \Phi^*(H^* \begin{bmatrix} \alpha_B \\ \ddot{q} \end{bmatrix} + a^*), \tag{A.13}$$

and

$$f^* = \Phi^{*T} B^* f + \Phi^{*T}(M^* \alpha^* + b^*) \tag{A.14}$$

Substituting equation (A.13) into equation (A.14) yields

$$H^{*T} f^* = \begin{bmatrix} f_B \\ \tau \end{bmatrix} = H^{*T}\Phi^{*T}B^* f + H^{*T}\Phi^{*T}\big(M^*\Phi^*(H^* \begin{bmatrix} \alpha_B \ddot{q} \end{bmatrix} + a^*) + b^*\big). \tag{A.15}$$

Simplification and combining block matrices conveniently yields

$$\begin{bmatrix} f_B \\ \tau \end{bmatrix} = \begin{bmatrix} \Phi_{nB}^T \Phi_{n+1,n}^T \\ H^T \Phi^T B \end{bmatrix} f + H^{*T}\Phi^{*T}\big(M^*\Phi^*(\begin{bmatrix} \alpha_B \\ H\ddot{q} + a \end{bmatrix}) + b^*\big),$$

$$\begin{bmatrix} f_B \\ \tau \end{bmatrix} = \begin{bmatrix} \Phi_{n+1,B}^T \\ H^T \Phi^T B \end{bmatrix} f + H^{*T}\Phi^{*T}\big(M^* \begin{bmatrix} \alpha_B \\ \hat{\Phi}_1 \alpha_B + \Phi H \ddot{q} + \Phi a \end{bmatrix} + b^*\big),$$

$$\begin{bmatrix} f_B \\ \tau \end{bmatrix} = \begin{bmatrix} \Phi_{n+1,B}^T \\ H^T \Phi^T B \end{bmatrix} f + H^{*T}\Phi^{*T}\big(\begin{bmatrix} M_B \alpha_B \\ M\hat{\Phi}_1 \alpha_B + M\Phi H \ddot{q} + M\Phi a \end{bmatrix} + b^*\big),$$

and

$$\begin{bmatrix} f_B \\ \tau \end{bmatrix} = \begin{bmatrix} \Phi_{n+1,B}^T \\ H^T \Phi^T B \end{bmatrix} f + \begin{bmatrix} (M_B + \hat{\Phi}_2 M \hat{\Phi}_1)\alpha_B + \hat{\Phi}_2 M \Phi H \ddot{q} \\ H^T \Phi^T M \hat{\Phi}_1 \alpha_B + H^T \Phi^T M \Phi H \ddot{q} \end{bmatrix} + \begin{bmatrix} \hat{\Phi}_2 M \Phi a + b_B + \hat{\Phi}_2 b \\ H^T \Phi^T M \Phi a + H^T \Phi^T b \end{bmatrix}, \tag{A.16}$$

where

$$\hat{\Phi}_1 = \begin{bmatrix} \Phi_{1B} \\ \vdots \\ \Phi_{nB} \end{bmatrix}, \tag{A.17}$$

and

$$\hat{\Phi}_2 = \begin{bmatrix} \Phi_{1B}^T & \dots & \Phi_{nB}^T \end{bmatrix}. \tag{A.18}$$

Finally, we obtain

$$\begin{bmatrix} f_b \\ \tau \end{bmatrix} = \begin{bmatrix} M_b & M_{rb} \\ M_{br} & M_r \end{bmatrix} \begin{bmatrix} \alpha_B \\ \ddot{q} \end{bmatrix} + \begin{bmatrix} C_B \\ C_r \end{bmatrix} + \begin{bmatrix} \Phi_{n+1,B}^T \\ J^T \end{bmatrix} f, \tag{A.19}$$

where

$$
\begin{aligned}
M_b &= M_B + \hat{\Phi}_2 M \hat{\Phi}_1, \\
M_{rb} &= \hat{\Phi}_2 M \Phi H, \\
M_{br} &= H^T \Phi^T M \hat{\Phi}_1, \\
M_r &= H^T \Phi^T M \Phi H, \\
C_B &= \hat{\Phi}_2 M \Phi a + b_B + \hat{\Phi}_2 b, \\
C_r &= H^T \Phi^T M \Phi a + H^T \Phi^T b.
\end{aligned}
$$

Note that the well-known manipulator Jacobian is given by $J = H^T \Phi^T B$. Similarly, M_r and C_r are the mass-inertia matrix and Centrifugal/Coriolis terms obtained for a standard fixed-base manipulator arm (Jain, 2010).
